# Antimicrobial resistance—a threat to the world’s sustainable development

**DOI:** 10.1080/03009734.2016.1195900

**Published:** 2016-07-13

**Authors:** Dušan Jasovský, Jasper Littmann, Anna Zorzet, Otto Cars

**Affiliations:** ReAct Europe, Department of Medical Sciences, Uppsala University, Uppsala, Sweden

**Keywords:** Antibiotic resistance, antimicrobial resistance, sustainable development, sustainable development goals

## Abstract

This commentary examines how specific sustainable development goals (SDGs) are affected by antimicrobial resistance and suggests how the issue can be better integrated into international policy processes. Moving beyond the importance of effective antibiotics for the treatment of acute infections and health care generally, we discuss how antimicrobial resistance also impacts on environmental, social, and economic targets in the SDG framework. The paper stresses the need for greater international collaboration and accountability distribution, and suggests steps towards a broader engagement of countries and United Nations agencies to foster global intersectoral action on antimicrobial resistance.

## Introduction

Despite many proposals and initiatives in recent decades, the world has failed to keep pace with microbes becoming increasingly resistant to available treatments, so-called antimicrobial resistance (AMR). Any antibiotic use contributes to increasing the number of micro-organisms on which drugs have no effect. AMR is thus an unavoidable phenomenon that undermines the effectiveness of basic and modern medicine and is affecting people from birth to death.

**Figure:**
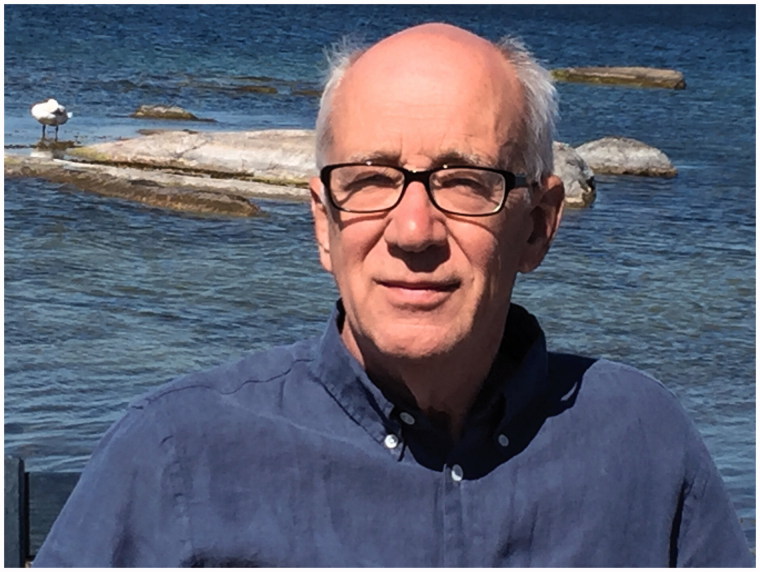
Otto Cars, winner of the Rudbeck Award 2015, at the Medical Faculty of Uppsala University for his long-term, persevering and successful strategic work in paying attention and taking measures to the increase of antibiotic resistance.

### ‘Post-antibiotic era’

Today, diminishing antimicrobial effectiveness represents a formidable threat to human and animal health and therefore to overall global development. Deaths from drug-resistant infections are projected to increase from currently 700,000 to 10 million annually, and cost estimates are as high as US$100 trillion worldwide by 2050 (1). Of special concern is the rapid global spread of multi-resistant bacteria, for some of which there is no available treatment. The prospect of the world entering a ‘post-antibiotic era’, where common infections can no longer be cured, is therefore a real possibility. Furthermore, unequal access to health care resources has meant that large populations in resource-poor settings never fully entered the antibiotic age to begin with. Consequently, universal access to effective antimicrobials represents one of the greatest opportunities to save and improve millions of lives each year (2,3).

### Global strategy

To address the challenge of AMR, the World Health Organization (WHO) first published a global strategy for its containment as far back as 2001 (4). However, insufficient human and financial resources to implement the strategy meant that its effect fell short of the intended goal. Over the past decade, the issue of AMR has become more prominent on the global health agenda. The increasing visibility of the consequences of AMR, both in terms of health outcomes and economic costs, has led to an increasing number of calls for global collective action to improve access to antimicrobial medicines on grounds of equity, to maintain their effectiveness, and to increase the supply of new products. Among the advocates of such action are heads of states and ministers as well as health care professionals and civil society organizations. But despite the growing recognition of the urgency of tackling AMR, international policies and global institutional arrangements are still lacking.

There have, however, been some positive developments lately. In 2015, the Global Action Plan on Antimicrobial Resistance was adopted at the 68th World Health Assembly in Geneva (5). Later that year, AMR was recognized as a threat to the world’s sustainability and development efforts in the resolution entitled ‘Transforming our world: the 2030 Agenda for Sustainable Development’, which incorporates the UN’s sustainable development goals (SDGs). Although omitted from specific SDG targets, AMR is included in paragraph 26 which states: ‘[W]e will equally accelerate the pace of progress made in fighting malaria, HIV/AIDS, tuberculosis, hepatitis, Ebola and other communicable diseases and epidemics, including by addressing growing antimicrobial resistance and the problem of unattended diseases affecting developing countries’ (6).

### The end of antibiotics

The startling truth is that for large populations the era of effective antibiotics has already, or will very soon, come to an end. Since AMR does not stop at national borders, it must be treated as a global challenge (7). Work must be undertaken to achieve a balance between supply and demand of urgently needed technologies through novel innovation models, health system strengthening, and massive efforts to change social norms (8). If allowed to take on pandemic proportions, AMR would not only risk undoing much of the progress made within the health sector, but also threaten other development achievements such as poverty reduction and economic growth (9).

Given the scale of the problem of AMR and the many areas of society affected by its consequences, solutions to the challenge we face must follow an integrated and comprehensive policy approach. AMR must be redefined in a broader context beyond human health, including agricultural and environmental issues, as well as health security, all within the framework of sustainable development, and should also be reflected in the implementation of the SDGs (10,11). Analogies with other fundamental global concerns, such as climate change, can help us understand the actual scope and irreversible consequences we face if decisive and far-reaching actions are not taken. The remainder of this paper will discuss how AMR may impact the achievement of specific SDGs.

## Implications for SDG 3

### SDG 3: Ensure healthy lives and promote well-being for all at all ages

*Maternal and child health*. Several of the adopted targets in the health-dedicated SDG 3 will be impossible to achieve without the availability of effective antibiotics. Their central role for the provision of maternal and child health has been recognized in the Every Woman Every Child initiative, which included antibiotics in a list of 13 life-saving commodities (12) that contributed to the achievement of the millennium development goals (MDGs) on maternal and child health. Especially in low- and middle-income countries (LMICs), where the maternal mortality rate is up to 19 times higher than in the rest of the world, the emergence of AMR currently jeopardizes the progress made in reducing maternal mortality over the previous 15 years (9).

*Communicable diseases*. Alarmingly, 214,000 neonatal sepsis deaths annually are directly attributable to drug-resistant pathogens (13). This severely impacts on plans to reduce neonatal mortality to less than 12 per 1,000 live births under SDG target 3.2 (6). In the last 15 years, child mortality has declined by 47%, which is largely due to reductions in deaths from pneumonia, a leading cause of child mortality globally (14). If drug resistance continues to rise, and if access to effective antibiotics is not guaranteed, these health gains could be reversed.

Sexually transmitted infections (STIs) are a major cause of disease globally, with an estimated 500 million new infections every year, causing long-term disability, infertility, and even death (15,16). Some of these infections are becoming increasingly drug-resistant. With approximately 106 million newly infected individuals every year, gonorrhoea is one of the most common STIs (16,17). It is therefore extremely worrying that some cases of gonorrhoea have become untreatable because of resistance to last-resort antibiotics (18). Furthermore, untreated STIs are also well-known risk factors for HIV transmission (19).

Attention should also be paid to emerging resistance to treatments for HIV, tuberculosis (TB), and malaria, which will create significant obstacles for the achievement of SDG target 3.3 (6). In the case of TB, half a million people developed multidrug-resistant TB in 2014, with an estimated death toll of 210,000. Extensively drug-resistant TB has been reported by 100 countries (20). In sub-Saharan Africa, drug resistance to commonly used HIV medicines was detected in almost 60% of cases (21), while drug-resistant malaria is on the rise in Southeast Asia (22).

In summary, drug-resistant pathogens could reverse the recent positive trend of falling global mortality rates from infectious diseases, which have decreased from 23% to 17% of total deaths over the last 15 years (9,23,24).

*Non-communicable diseases*. The impact of AMR is not limited to infectious diseases. It also has potentially disastrous consequences for the treatment of many non-communicable diseases (NCDs). A clear example is the threat that AMR poses to the safety and effectiveness of procedures such as surgical interventions, cancer treatment, and organ transplants (8). For example, up to 50% of pathogens causing surgical site infections are resistant to standard prophylactic antibiotics in the US. Similarly, every fourth patient on cancer treatment suffered from infections caused by pathogens that were resistant to commonly used antibiotics (25). Immunocompromised patients who have undergone transplantations or chemotherapy (26) or who have AIDS are more vulnerable to infections (27). Access to effective antibiotics is therefore vital to meeting several SDG targets on NCDs.

*Universal health coverage*. Given the disproportionate global distribution of infectious diseases, with the heaviest burden in LMICs, the emergence of AMR creates bottlenecks for establishing universal health coverage globally, which SDG target 3.8 (6) calls for. Arguably, no health system will be sustainable without effective antibiotics (28), as demonstrated by an example where the cost of treating and caring for a single patient with extensively drug-resistant tuberculosis has been estimated at a staggering $482,000 in the US (29). The indisputable, yet difficult-to-measure magnitude with which antimicrobial medicines of lowered quality contribute to global AMR rates should also be addressed accordingly as part of an integrated solution (10).

## Implications for other SDGs

### SDG 1: End poverty in all its forms everywhere

From an international development perspective, AMR strikes hardest at the poor. Rates of resistant bacteria are high, and affordable treatment options are often not available, especially since many patients pay for medicines out-of-pocket. Moreover, the spread of diseases is enabled by weak sanitary systems (8). Treating drug-resistant infections is costlier, takes longer, and has lower chances of success than treating drug-susceptible infections. As a result of these factors, AMR negatively impacts on national economic performance (30), which could ultimately contribute to slowing down progress towards SDG 1.

### SDG 2: End hunger, achieve food security and improved nutrition, and promote sustainable agriculture

The rapid growth of intensive production systems as a response to increased demand for animal protein has been estimated to increase the consumption of antimicrobials in the food animal sector by two-thirds between 2010 and 2030 (31,32). This inherent conflict has to be resolved if we are to meet the indicators of SDG 2 to double the agricultural productivity by 2030 and to ensure the implementation of sustainable food production systems and resilient agricultural practices.

To show the implications of agricultural use of antibiotics, researchers recently studied soil samples spanning 100 years. The study showed that levels of antibiotic resistance genes started to increase by the mid-1970s in soils fertilized by manure. The change in types of resistance genes also roughly paralleled the first reports of these genes in clinical isolates from hospitals. Perhaps even more interestingly, the level of some resistance genes decreased again following a ban on non-therapeutic antibiotic use, suggesting accumulated soil resistance genes could be reduced by antibiotic stewardship efforts (33).

To develop sustainable animal production practices where high productivity is reached without inappropriate use of antimicrobials is of crucial importance (34). There are several examples of high productivity being reached without use of antibiotics for growth promotion or routine mass-medication, and without severe economic losses (35). The key elements here are good animal husbandry practices that prevent disease, combined with commercial disincentives and a legal framework that regulates the use of antibiotics in the animal sector.

### SDG 6: Ensure availability and sustainable management of water and sanitation for all

Waste products from hospitals, antibiotic manufacturing plants, and agriculture contribute to increased amounts of antibiotic residues and resistant bacteria in aquatic ecosystems (36,37). For example, measurements at a wastewater plant receiving waste from approximately 90 drug manufacturers showed that 45 kg of a common antibiotic were released into a nearby river each day. The concentration of the antibiotic in the river was higher than it would be in a patient who was treated with the drug (38). As antibiotic residues can be carried by water and through sediments and soil, gradients of different antibiotic concentrations will form, and even very low antibiotic concentrations may be enough to select for highly resistant bacteria (39). Importantly, lack of access to clean water and sanitation also facilitates the spread of bacterial diseases, leading to increased morbidity and mortality, especially in children.

### SDG 8: Promote sustained, inclusive, and sustainable economic growth, full and productive employment, and decent work for all

As mentioned, resistance to antimicrobial drugs already causes an estimated 700,000 deaths annually and—without effective action—is predicted to cause 10 million deaths annually and cost up to US$100 trillion by 2050 (1). Losing effective antibiotics for the treatment of infectious diseases adds an extra burden to states’ health expenditure and can lower productivity, household income, and tax revenues and lead to losses in GDP (30). Such enormous costs may undermine efforts for sustainable economic growth as emphasized in SDG 8, making AMR a critical global development issue (1,30).

### SDG 12: Ensure sustainable consumption and production patterns

Antibiotics must be looked upon as a scarce and potentially non-renewable global resource. Ensuring sustainable consumption and production of this resource, and recognizing the linkages between SDG 12 and other goals such as SDG 2, requires a multipronged approach that addresses three fundamental issues.

First, access to effective antibiotics could be considered an integral part of the fulfilment of the human right to health. Globally, a staggering 5.7 million people die each year from treatable infectious diseases due to lack of access to antibiotics (2). An estimation shows that universal provision of antibiotics could avert approximately 445,000 community-acquired pneumonia deaths in children under five years of age (13).

Second, constructing a sustainable yet accessible model of antibiotic distribution and consumption requires conservation mechanisms that would reduce the need for antibiotics while ensuring their appropriate use (40). This would among other things include evidence-based rational use programmes and strong surveillance schemes, coupled with a responsive international framework that includes means of implementation for low-resource countries and locally tailored regulations (2).

Third, greater efforts to promote the innovation of new antimicrobials and diagnostics are needed (41). The research and development of novel antibiotics has for long been de-prioritized due to scientific and financial bottlenecks (42). The latest class of antibiotics was discovered in 1987 (43), and there is a dangerous shortfall of new drugs in the pipeline. Considering the enormous global public value of antibiotics, the failed innovation model needs to be driven by public health needs and transformed to include, for example, public–private projects or novel business models as presented in SDG 9.

### SDG 17: Strengthen the means of implementation and revitalize the Global Partnership for Sustainable Development

Global sustainability efforts require partnerships between governments, civil society, and the private sector as called for in the final SDG 17. Framing AMR as a sustainability issue will therefore urgently require policy and institutional coherence at all policy levels, from global to local (44). The responsibility of governments and their international co-operation constitutes the primary Global Partnership as reaffirmed in SDG 17.

### Multi-stakeholder partnerships

To avoid an AMR ‘tragedy of the commons’ situation, antimicrobial effectiveness needs to be recognized as a fundamentally important global public good and governed accordingly. In addition to the Global Action Plan on AMR, there is critical need for cross-sectoral global action by countries and other stakeholders to complement it. There have been multiple calls from various bodies including the G7 Health Ministers, the Foreign Policy and Global Health Initiative, and an alliance of champion countries for a multi-stakeholder approach to control AMR. Such an approach could help to ensure that all relevant aspects of AMR are addressed through intergovernmental and cross-UN agency action based on respective expertise. To successfully initiate and manage such a multi-stakeholder process, the discussion of global co-ordination and leadership redistribution must be initiated early on. It is also vital to consider and emulate lessons learned from comparable global political initiatives in global health and other areas. Examples of such initiatives include the global response to the AIDS crisis where the UN General Assembly adopted the Declaration of Commitment on HIV/AIDS and the High-Level Meeting on the Prevention and Control of Non-Communicable Diseases (NCDs) that helped to chart a new international agenda on NCDs. Similarly, the global response to climate change has shown the type of multi-stakeholder partnerships needed to contain AMR.

### Mobilized means of implementation for technology and capacity building

Political will needs to be reflected in significant commitments to mobilizing means of implementation for technology and capacity building for both LMICs and high-income countries. Here, the role of national actors spanning from governmental entities to UN agencies with strong national presence is essential in the process of effective implementation. It is likewise crucial to strengthen knowledge sharing to support access to innovations in the field of AMR. These actions must be coupled with international support for implementing effective and targeted capacity building in LMICs to support national plans to implement all the sustainable development goals, including national action plans on AMR.

### Data, monitoring, and accountability

Financing such national action plans, especially in LMICs, would consequently enable monitoring and accountability mechanisms for data on access to antibiotics, their use and resistance levels, which are key to lasting global collective action.

## Summary and conclusions

AMR is not a disease for which we should expect ultimately to develop a cure. Instead it is a silent pandemic that is here to stay. AMR is a phenomenon where micro-organisms adapt through evolution to survive the onslaught of antimicrobial drugs. It undermines the treatment of many common diseases as well as standard surgical procedures. As we have shown above, it is also a challenge to global development at large. Antimicrobial effectiveness must be looked upon as a limited global public good on the verge of becoming scarce, and the world has a collective responsibility to preserve it in order to avoid countless future victims of drug-resistant infections (45). There is no single ‘silver bullet’ to address AMR. We need an adaptive, multipronged approach that operates across the SDGs, involving many stakeholders. Accordingly, we cannot merely look to the biomedical or health sectors for solutions. AMR is a systems failure that requires a cross-sectoral response. By placing AMR within the SDG agenda, we seek to intensify and widen the international political commitment to finding a solution to this emerging threat before it turns into a global crisis.
